# m^6^A reader IGF2BP2-stabilized CASC9 accelerates glioblastoma aerobic glycolysis by enhancing HK2 mRNA stability

**DOI:** 10.1038/s41420-021-00674-y

**Published:** 2021-10-13

**Authors:** Hongjiang Liu, Shan Qin, Changqi Liu, Le Jiang, Chen Li, Jiankai Yang, Shunyao Zhang, Zhongjie Yan, Xiaopeng Liu, Jipeng Yang, Xiaofeng Sun

**Affiliations:** 1 Department of Neurosurgery, The Second Hospital of Hebei Medical University, 050000 Shijiazhuang, Hebei China; 2 Department of Cardiology, The Second Hospital of Hebei Medical University, 050000 Shijiazhuang, Hebei China; 3 Medical Records Room, The Second Hospital of Hebei Medical University, 050000 Shijiazhuang, Hebei China; 4 Office of Academic Research, The Second Hospital of Hebei Medical University, 050000 Shijiazhuang, Hebei China

**Keywords:** CNS cancer, Cancer metabolism

## Abstract

N^6^-methyladenosine (m^6^A) has been identified to exert critical roles in human cancer; however, the regulation of m^6^A modification on glioblastoma multiforme (GBM) and long non-coding RNA (lncRNA) CASC9 (cancer susceptibility 9) is still unclear. Firstly, MeRIP-Seq revealed the m^6^A profile in the GBM. Moreover, the m^6^A-related lncRNA CASC9 expression was significantly elevated in the GBM tissue and its ectopic high expression was associated with poor survival, acting as an independent prognostic factor for GBM patients. Functionally, the aerobic glycolysis was promoted in the CASC9 overexpression transfection, which was inhibited in CASC9 knockdown in GBM cells. Mechanistically, m^6^A reader IGF2BP2 (insulin-like growth factor 2 mRNA binding protein 2) could recognize the m^6^A site of CASC9 and enhance its stability, then CASC9 cooperated with IGF2BP2, forming an IGF2BP2/CASC9 complex, to increase the HK2 (Hexokinase 2) mRNA stability. Our findings reveal that CASC9/IGF2BP2/HK2 axis promotes the aerobic glycolysis of GBM.

## Introduction

Glioblastoma multiforme (GBM) is one of the most aggressive tumors of the central nervous system while representing 80% of all malignant brain tumors [1, 2].

Since decades GBM is characterized by a median overall survival of 15 months, suggesting the tremendous hazard of GBM [3, 4]. Although GBM is very dangerous, it is hardly excised by surgical treatment or intensive treatments, leading to a low survival rate [5]. GBM is characterized by a median overall survival around 15 months for decades, suggesting an urgent need for an accurate underlying mechanism of GBM.

N^6^-methyladenosine (m^6^A) is the most abundant modification in mRNA mediated by m^6^A methyltransferases, demethylases and readers [6]. M^6^A methyltransferases install the m^6^A modification on RNA, especially methyltransferase-like3 (METTL3) and its auxiliary partners METTL14 and WTAP. Currently, m^6^A modification is found to be involved in most pivotal biological processes, including stem cells differentiation, spermatogenesis, RNA metabolism and so on. For example, m^6^A demethylase ALKBH5 is highly expressed in GBM stem-like cells and ALKBH5 mediated the demethylation of FOXM1 nascent transcripts. Moreover, lncRNA FOXM1-AS accelerates the interaction of ALKBH5 and FOXM1 nascent transcripts, leading to the FOXM1 expression increasing [7]. Moreover, researchers found that the m^6^A level is decreased in glioma tissue and the high level of m^6^A results in a decreased migration and proliferation ability [8]. Therefore, these findings reveal the potential roles of m^6^A on GBM.

More than the DNA methylation, histone and chromatin modifications, m^6^A is one of the most researched hotspots in epigenetics. IGF2BP2 is one of the main RNA reader regulating human tumorigenesis. For example, upregulation of IGF2BP2 is correlated to pancreatic cancer patients’ poor outcomes. Moreover, IGF2BP2 positively regulates lncRNA DANCR expression [9]. Here, our research paid close attention to the coordination within m^6^A and lncRNA CASC9 (cancer susceptibility 9) based on the MeRIP-Seq (methylated RNA immunoprecipitation sequencing) in GBM. CASC9 cooperated with IGF2BP2 (insulin-like growth factor 2 mRNA binding protein 2) to increase the HK2 (Hexokinase 2) mRNA stability, thereby promoting aerobic glycolysis.

## Results

### MeRIP-Seq revealed the m^6^A profile in the GBM

**Fig. 1:**
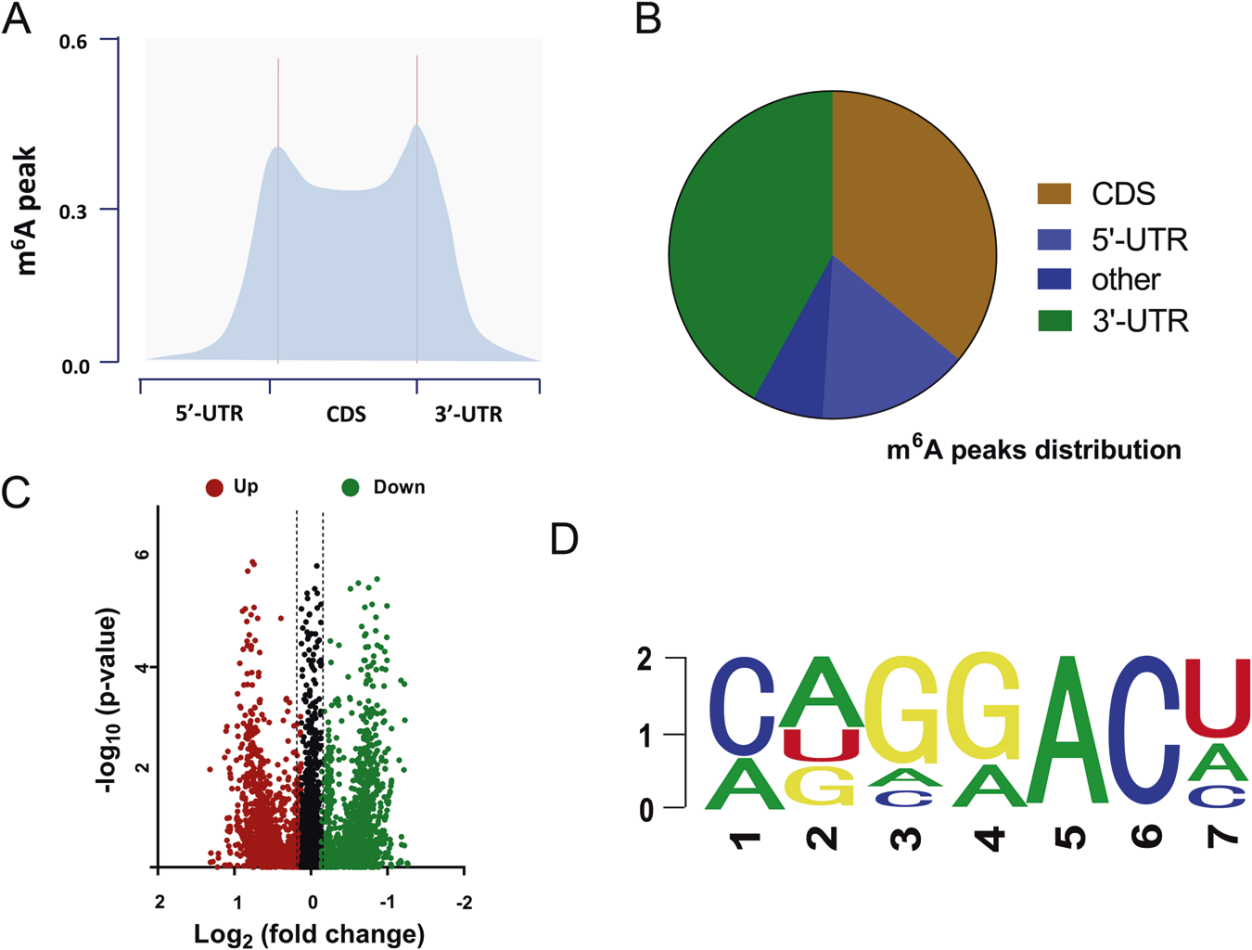
MeRIP-Seq revealed the m^6^A profile in the GBM. **A** MeRIP-Seq was performed and found that the m^6^A peaks frequency was mainly located in the 5-end untranslated regions (5ʹ-UTR), coding region (CDS) and 3ʹ-UTR. **B** Pie chart demonstrated the distribution of m^6^A peaks in the GBM genome. **C** Volcano plot displayed the upregulated or downregulated targets labeled with m^6^A peaks. **D** The m^6^A motif with the highest frequency is the GGACU motif.

To explore the m^6^A profile in the GBM, MeRIP-Seq was performed and detected. It is found that the m^6^A peaks frequency was mainly located in the 5-end untranslated regions (5ʹ-UTR), coding region (CDS) and 3ʹ-UTR (Fig. 1A). The pie chart demonstrated the distribution of m^6^A peaks in the GBM genome (Fig. 1B). The volcano plot displayed the upregulated or downregulated targets labeled with m^6^A peaks (Fig. 1C). Among the m^6^A motif, the GGACU motif occupied the main ingredient (Fig. 1D). Collectively, the above results implied that MeRIP-Seq revealed the m^6^A profile in the GBM.

### m^6^A modified lncRNA CASC9 indicated the unfavorable prognosis of GBM

**Fig. 2:**
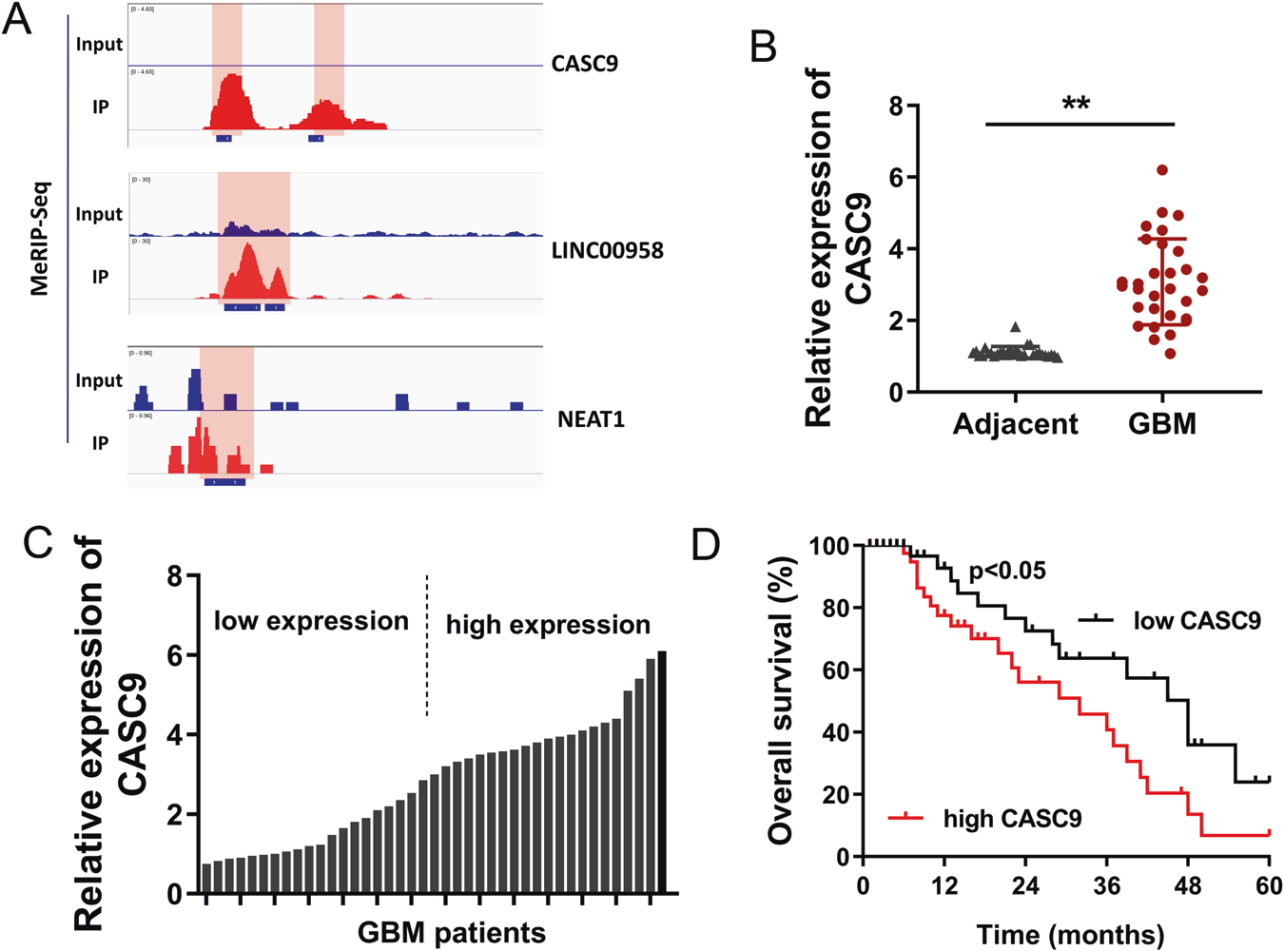
m^6^A modified lncRNA CASC9 indicated the unfavorable prognosis of GBM. **A** Several m^6^A modified lncRNAs were screened by MeRIP-Seq. The m^6^A modified sites in the RRACH motif were shown using IGV (Integrative Genomics Viewer). **B** In the clinically collected GBM tissue specimens, CASC9 expression was detected using RT-PCR as compared to the adjacent normal tissue. **C** On the basis of median value, the clinical tissue specimens were divided into high expression group and low expression group. **D** Overall survival (OS) curve calculated by the Kaplan−Meier method for GBM patients. Data are presented as the mean ± SD. **P < 0.01.

According to the data analysis of MeRIP-Seq, we found that several lncRNAs owned the m^6^A modified site in the RRACH motif. After screening, several m^6^A modified lncRNAs were selected and displayed (Fig. 2A). In these candidate RNAs, we focused on the lncRNA CASC9 and investigated its functions in the GBM tumorigenesis. Firstly, in the clinically collected GBM tissue specimens, CASC9 expression elevated when compared with the adjacent normal tissue (Fig. 2B). On the basis of median value, the clinical tissue specimens were divided into high expression group and low expression group (Fig. 2C). Overall survival (OS) curve calculated by the Kaplan−Meier method and survival for GBM patients showed that the patients who had high levels of CASC9 within tissues had remarkably shorter overall survival rate as compared to low levels of CASC9 (Fig. 2D). Clinically, CASC9 may serve as an independent factor to predict the prognosis of GBM patients. Collectively, the above results indicated that m^6^A modified lncRNA CASC9 indicated the unfavorable prognosis of GBM.

### m^6^A reader IGF2BP2 enhanced the stability of lncRNA CASC9

**Fig. 3:**
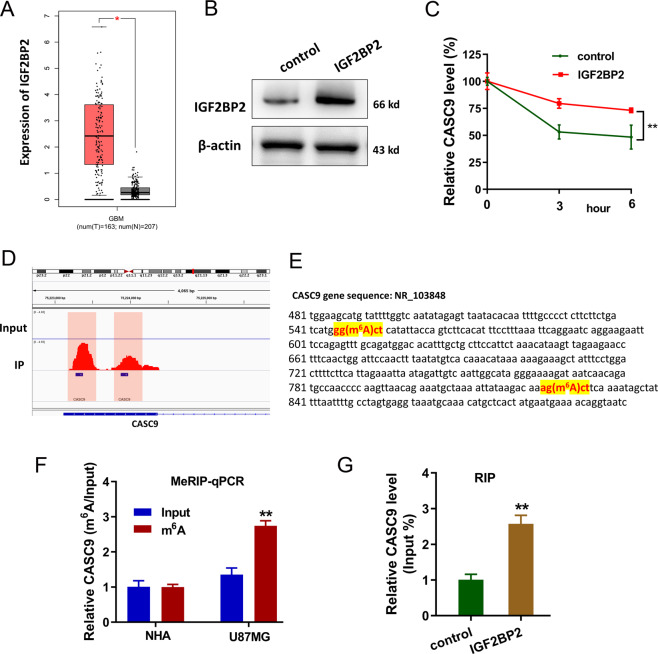
m^6^A reader IGF2BP2 enhanced the stability of lncRNA CASC9. **A** In the GBM cohort, IGF2BP2 was found to be upregulated according to the GEPIA dataset (http://gepia.cancer-pku.cn/index). **B** Western blot analysis found the IGF2BP2 expression in U87MG cells with IGF2BP2 overexpression by plasmids transfection. **C** RNA stability assay demonstrated the remaining lncRNA CASC9 in U87MG cells when treated with Act D. **D** MeRIP-Seq data by IGV showed the two m^6^A modification sites in the 3ʹ-UTR of CASC9. **E** The location of m^6^A modification sites in the CASC9 gene. **F** MeRIP-qPCR demonstrated the CASC9 expression in GBM cell lines (U87MG) using anti-m^6^A antibody. **G** RIP-qPCR showed the interaction within CASC9 and IGF2BP2 in U87MG cells using anti-IGF2BP2 antibody. Data are presented as the mean ± SD. **P < 0.01.

In the GBM cohort, IGF2BP2 was found to be upregulated according to the GEPIA dataset (http://gepia.cancer-pku.cn/index) (Fig. 3A). To evaluate the interaction of IGF2BP2 and CASC9, IGF2BP2 overexpression was established by plasmids transfection (Fig. 3B). RNA stability assay demonstrated that IGF2BP2 overexpression could enhance the stability of lncRNA CASC9 in U87MG cells when treated with Act D (Fig. 3C). MeRIP-Seq data were displayed by IGV and there were two m^6^A modification sites in the 3ʹ-UTR of CASC9 (Fig. 3D). Accurately, the two sites’ sequences were shown in the genomic location (Fig. 3E). MeRIP-qPCR demonstrated that CASC9 was enriched in the GBM cell lines (U87MG) using anti-m^6^A antibody (Fig. 3F). Moreover, RIP (RNA-immunoprecipitation)-qPCR showed that CASC9 could interact with the IGF2BP2 in U87MG cells (Fig. 3G). In conclusion, these findings suggested that m^6^A reader IGF2BP2, probably via its interaction with CASC9, enhanced the stability of lncRNA CASC9, thereby promoting CASC9 expression.

### LncRNA CASC9 accelerated the aerobic glycolysis of GBM

**Fig. 4:**
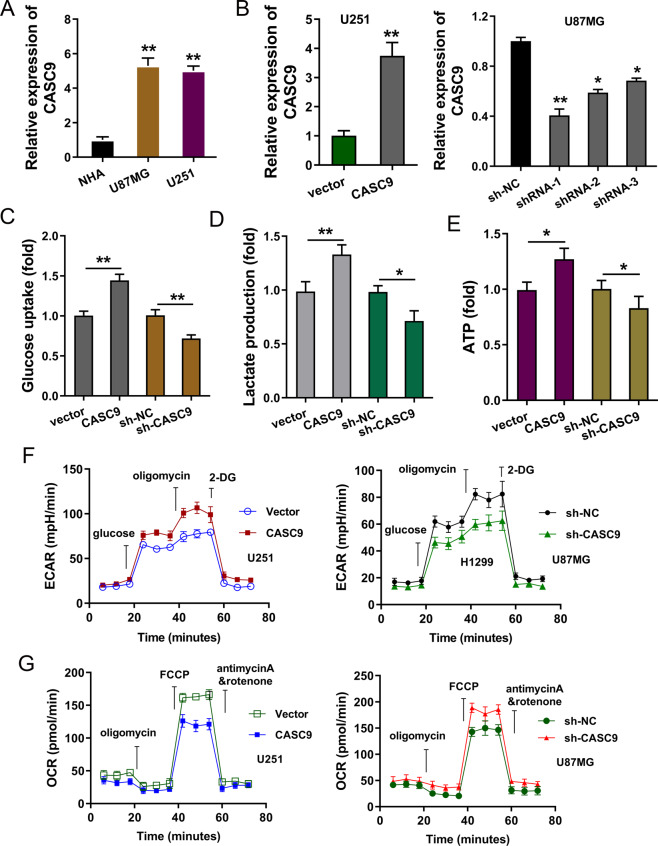
LncRNA CASC9 accelerated the aerobic glycolysis of GBM. **A** RT-PCR unveiled the CASC9 expression in human glioblastoma cell lines (U87MG, U251) relative to normal human astrocytes (NHA). **B** ShRNA-expressing lentiviruses targeting CASC9 were transfected into U87MG cells. CASC9 overexpression plasmids were transfected into U251 cells. RT-PCR unveiled the CASC9 expression relative to the control group. **C** Glucose uptake analysis, **D** lactate production and **E** ATP generation analysis were performed in U251 cells and U87MG cells to control group (vector or shRNA). **F** Extracellular acidification rate (ECAR) analysis was performed for the glycolysis stress test in U251 cells and U87MG cells. **G** Oxygen consumption rate (OCR) assay was performed for the respiratory rate in U251 cells and U87MG cells. Data are presented as the mean ± SD. **P < 0.01, *P < 0.05.

Firstly, CASC9 expression was elevated in the GBM cells (U251, U87MG) as compared to normal cells (Fig. 4A). To test whether CASC9 was essential for GBM cellular phenotype, we silenced the expression of CASC9 in GBM cells through shRNA-expressing lentiviruses in U87MG cells, and CASC9 overexpression was constructed in U251 cells through plasmids transfection (Fig. 4B). Glucose uptake analysis, lactate production and ATP generation analysis demonstrated that CASC9 overexpression promoted the glycolytic capacity, including glucose uptake (Fig. 4C), lactate production (Fig. 4D) and ATP generation (Fig. 4E). Meanwhile, CASC9 knockdown repressed the glycolytic capacity. In further research, extracellular acidification rate (ECAR) analysis found that CASC9 overexpression promoted the ECAR in U251 cells, and CASC9 knockdown reduced ECAR in U87MG cells (Fig. 4F). Besides, oxygen consumption rate (OCR) assay revealed that CASC9 overexpression promoted respiratory rate in U251 cells, and CASC9 knockdown reduced respiratory rate in U87MG cells (Fig. 4G). Taken together, the data suggest that CASC9 accelerated the aerobic glycolysis of GBM.

### CASC9/IGF2BP2 complex contributed to the stability of HK2 mRNA

**Fig. 5:**
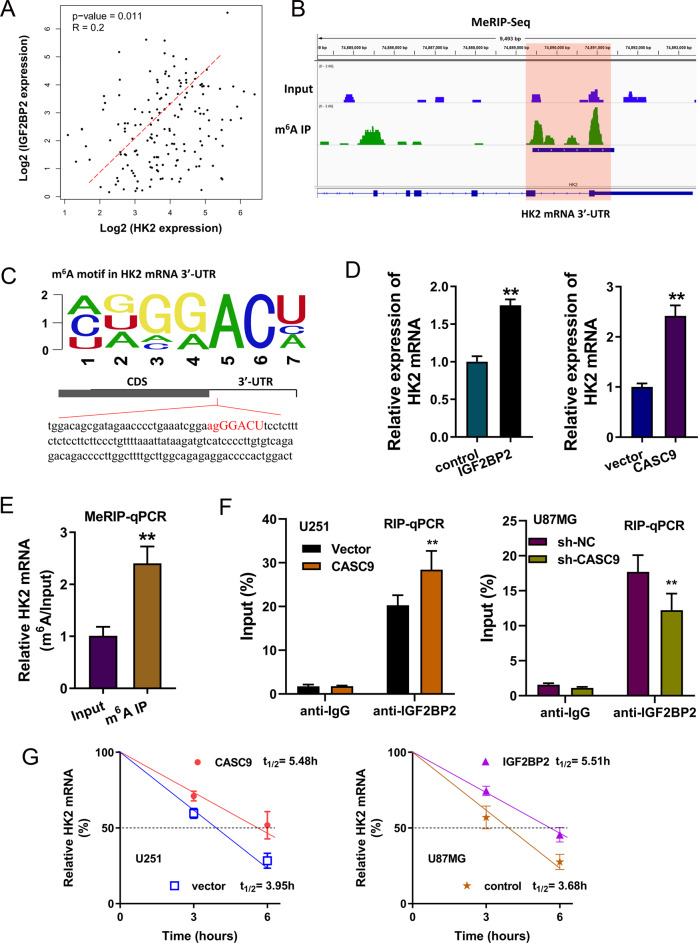
CASC9/IGF2BP2 complex contributed to the stability of HK2 mRNA. **A** The correlation analysis within the expression of IGF2BP2 and HK2 in the GBM group, which was based on ATGC (http://gepia.cancer-pku.cn/). **B** MeRIP-Seq indicated that there was a remarkable m^6^A site in the 3ʹ-UTR of HK2 mRNA. **C** The m^6^A site in the 3ʹ-UTR of HK2 mRNA was AGGGACU. **D** In U251 cells, RT-PCR assay detected the HK2 mRNA levels when transfected with IGF2BP2 overexpression. In U87MG cells, RT-PCR assay detected the HK2 mRNA levels when transfected with CASC9 overexpression. **E** MeRIP-PCR assay illustrated the HK2 mRNA expression using anti-m^6^A antibody as compared to Input in U87MG. **F** RIP-qPCR unveiled the HK2 mRNA level in U251 cells or U87MG cells using IGF2BP2 antibody. **G** RNA stability assay demonstrated the HK2 mRNA in U251 cells or in U87MG cells when treated with Act D. Data are presented as the mean ± SD. **P < 0.01.

In the correlation analysis, we found that the expression of IGF2BP2 was positively correlated to the expression of HK2 in the GBM group (Fig. 5A) based on the public database data (GEPIA, http://gepia.cancer-pku.cn/). According to the MeRIP-Seq, there was a remarkable m^6^A site in the 3ʹ-UTR of HK2 mRNA (Fig. 5B). Consistent with our original hypothesis, the m^6^A site in the 3ʹ-UTR of HK2 mRNA was identified to be AGGGACU (Fig. 5C). RT-PCR assay demonstrated that the HK2 mRNA level was increased in the IGF2BP2 overexpression transfection (U251 cells) and in the CASC9 overexpression transfection (U87MG cells) (Fig. 5D). MeRIP-PCR assay illustrated that HK2 mRNA was enriched in the anti-m^6^A antibody group as compared to the Input group (Fig. 5E). RIP-qPCR unveiled that CASC9 overexpression promoted the interaction within IGF2BP2 and HK2 mRNA, while CASC9 silencing reduced the combination within IGF2BP2 and HK2 mRNA (Fig. 5F). RNA stability assay demonstrated that CASC9 overexpression promoted the stability of HK2 mRNA in U251 cells. Moreover, IGF2BP2 overexpression could enhance the stability of HK2 mRNA in U87MG cells when treated with Act D (Fig. 5G). In conclusion, these results suggested that the function of the CASC9/IGF2BP2 complex in regulating HK2 mRNA depended on its binding to the HK2 m^6^A modification site.

## Discussion

Recent evidence demonstrated that m^6^A is an intense research field and exerts critical functions on human cancers, encompassing GBM [10–12]. It is known that m^6^A could regulate the metabolism of RNA, including mRNA, miRNA and lncRNAs [13, 14]. However, the potential role of m^6^A and lncRNA in GBM is still hazy.

In the data of MeRIP-Seq, we found that hundreds of potential RNA owned the m^6^A modification sites. Moreover, m^6^A peaks frequency was mainly located in the 5-end untranslated regions (5ʹ-UTR), coding region (CDS) and 3ʹ-UTR. Upregulated or downregulated targets labeled with m^6^A peaks were identified. Among the m^6^A motif, the GGACU motif occupied the main ingredient. Interestingly, we found that there was an m^6^A site in the lncRNA CASC9. On account of the regulation of m^6^A on RNA metabolism, m^6^A key enzyme may function as a regulator for CASC9.

In the clinical analysis, we found that the m^6^A modified lncRNA CASC9 was upregulated in GBM and indicated the unfavorable prognosis of GBM [15]. Clinically, CASC9 may serve as an independent factor to predict the prognosis of GBM patients. In the functional assays, using CASC9 overexpression transfection and CASC9 silencing, we found that CASC9 could positively regulate the aerobic glycolysis in GBM cells. Under basal conditions, there was no difference on glucose uptake, lactate production and ECAR. Aerobic glycolysis, also known as the Warburg effect, functions as an essential initiating factor for GBM [16–18]. Aerobic glycolysis not only supports energy production but also provides the carbon skeletons for the cellular synthesis of nucleic acids in tumor cells. Besides, fatty acids act as the energetic substrates or materials for lipid membrane construction. Therefore, the energy and outcomes of aerobic glycolysis could remarkably promote the development and progression of GBM.

Another valuable finding is that the m^6^A-modified CASC9 expression was mediated by the specific m^6^A reader IGF2BP2. IGF2BP2 has been found to be upregulated in glioma and indicates a poor prognosis [19]. MeRIP-Seq displayed that there were two m^6^A modification sites in the 3ʹ-UTR of CASC9. RNA stability assay and RT-PCR demonstrated that IGF2BP2 overexpression could enhance the stability of lncRNA CASC9 and promote its expression. Moreover, RIP illustrated that m^6^A reader IGF2BP2 significantly interacted with CASC9, highlighting a molecular interaction within IGF2BP2 and CASC9.

**Fig. 6:**
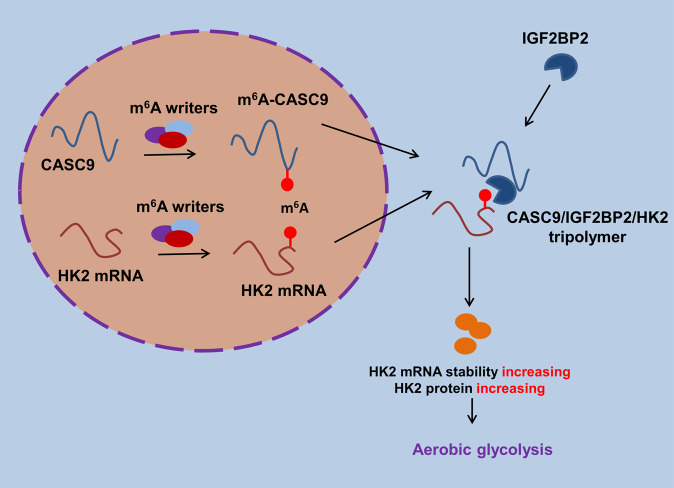
Schematic diagram of CASC9 on the GBM aerobic glycolysis through IGF2BP2/HK2 pathway.

Given that CASC9 could regulate aerobic glycolysis, we tried to investigate the potential downstream targets of CASC9. After screening, we found that there was an obvious m^6^A site in the 3ʹ-UTR of HK2 mRNA. Glycolytic enzyme hexokinase 2 (HK2) is a crucial regulator for the GBM Warburg effect [20, 21]. Functional analysis found that the overexpression of CASC9 could enhance the interaction within IGF2BP2 and HK2 mRNA. However, depleting CASC9 disrupted the interaction within IGF2BP2 and HK2 mRNA. RNA stability assay demonstrated that CASC9 overexpression promoted the stability of HK2 mRNA. Moreover, IGF2BP2 overexpression could enhance the stability of HK2 mRNA when treated with Act D. In public database data (GEPIA, http://gepia.cancer-pku.cn/), IGF2BP2 expression is positively correlated to HK2; however, the correlation coefficient of IGF2BP2 and HK2 is not so significant (0.2). In conclusion, these results suggested that the function of the CASC9/IGF2BP2 complex in regulating HK2 mRNA depended on its binding to the HK2 m^6^A modification site (Fig. 6).

In conclusion, we describe the function of m^6^A and lncRNA in the GBM aerobic glycolysis. CASC9/IGF2BP2 complex positively regulates the HK2 mRNA, which depended on its binding to the HK2 m^6^A modification site. Our findings uncover a critical function for m^6^A-modified lncRNA CASC9 and provide insight into a critical role of m^6^A methylation in GBM.

## Materials and methods

### Brain tissue collection

Both normal brain tissue and GBM tissue were collected during surgical specimens at The Second Hospital of Hebei Medical University (Table 1). All these processes were performed in accordance with the protocols approved by The Second Hospital of Hebei Medical University Ethics Committee in compliance with the Declaration of Helsinki. Written informed consents were obtained from all study participants before the procedure with an explanation of experimental details.

**Table 1:** Relationship between CASC9 expression and clinicopathological characteristics of GBM patients.

Variable	Total = 30	CASC9 expression		p
		Low = 15	High = 15	
Gender				
Male	17	9	8	0.768
Female	13	6	7	
Age				
<45	11	5	6	0.364
≥45	19	10	9	
Tumor size				
<3 cm	10	5	5	0.561
3−5 cm	16	8	8	
>5 cm	4	2	2	
WHO grading				
I−II	13	5	8	0.043*
III−IV	17	10	7	
KPS				
≥80	19	10	9	0.427
<80	11	5	6	

*KPS* Karnofsky performance score, *WHO* World Health Organization.

*P < 0.05 represents statistical differences.

### Cell culture

Normal human astrocytes (NHA) and human glioblastoma cell lines (U87MG, U251) were purchased from the American Type Culture Collection (ATCC, USA). Cells were cultured in 1640 medium (Gibco, USA) supplemented with 10% FBS (Gibco, USA), 100 U/ml penicillin and streptomycin (Gibco, USA) in humidified 37 °C and 5% CO_2_ incubator (Thermo, Germany).

### shRNA-lenti-viral construction and infection

Independent recombinant lentiviruses (pCDH-CMV-MCS-GFP) shRNA targeting CASC9 for stable knockdown were constructed by Shanghai Genechem Co., Ltd., (Shanghai, China). Stably transfected cells were chosen with 1 μg/ml puromycin (Calbiochem, USA) for 2 weeks. Overexpression CASC9 full length was synthesized and cloned into a pLKO.1-derived plasmid vector. The other siRNAs were bought by Riobo Bio (Guangzhou, China) and transfected into GBM cells using Lipofectamine 2000 (Invitrogen) when cells were grown to 70% confluent.

## Supplementary information

Supplementary file 1

Supplementary data

Table S1

## Data Availability

Methods details are shown in Supplementary file 1.
