# Supplementary material for: m6A reader IGF2BP2-stabilized CASC9 accelerates glioblastoma aerobic glycolysis by enhancing HK2 mRNA stability
Source: Cell Death Discov. 2021 Oct 13;7:292. doi: 10.1038/s41420-021-00674-y (PMC8514511; doi:10.1038/s41420-021-00674-y)
Supplement: Supplementary file 1 [file 41420_2021_674_MOESM1_ESM.docx]

**Supplementary file 1：**

**RNA preparation and quantitative real-time PCR**

RNA from GBM cell and tissue specimens were extracted using rapid RNA extraction kit (QIAGEN, Hilden, Germany) and then cDNA was reversely transcribed using SuperScript First-Stand Synthesis system (Invitrogen, USA). RNA relative expressions were evaluated by quantitative real-time PCR using SYBR Premix Taq (Applied Biosystems, US) on Applied Biosystems 7500. Data results of transcript levels were analyzed using 2^−△△Ct^ method. All primers were listed in additional **Table S1**.

**m^6^A methylated RNA immunoprecipitation sequencing (MeRIP-Seq)**

MeRIP-seq and data analysis were performed as described previously([22](#_ENREF_22)). In brief, total RNAs were extracted from GBM (U87MG) cells and normal cells and thenreverse-transcribed to cDNA. RNA-seq library was constructed using purified RNA. Specific m^6^A antibody was used to immunoprecipitate RNA. Both m^6^A IP sample group and the input sample group were conveyed to single-end sequencing on an Illumina NextSeq 500 sequencer. RNAs sites (peaks) with methylation on Input or m^6^A IP group were identified using MACS software. The sequencing data was displayed using IGV tool (Integrative Genomics Viewer).

**Western blot**

The protein lysate from the GBM cells was prepared according to manufacturer’s protocols. Protein supernatants was extracted using StrataClean Resin (Agilent Technologies) and detected using the BCA protein assay kit (KeyGEN). The separated protein was transferred onto PVDF membranes (Millipore, USA) and then blocked in TBS-T containing 5% BSA at room temperature for 1 h. Antibody for IGF2BP2 was purchased from Abcam (USA) and diluted in 1:1000 dilution ad then incubated with PVDF members overnight at 4 °C. Membranes were then incubated with secondary horseradish peroxidase-conjugated antibodies at room temperature and detected using ECL system (GE Healthcare) and ImageJ software.

**Glucose,** **lactate, ATP analysis, extracellular acidification rate (ECAR) and** **oxygen consumption rate (OCR)**

The glucose uptake, lactate production and ATP generation level were respectively measured using colorimetric glucose assay kit (BioVision, Milpitas, CA, USA), Lactate Colorimetric Assay Kit (BioVision) and ATP assay kit (Beyotime). For glycolysis stress test, the ECAR was detected using Seahorse XF 96 Extracellular Flux Analyzer (Seahorse Bioscience, Agilent Technologies, Santa Clara, CA, USA). For respiratory rate, OCR was detected using Seahorse XF Cell Mito Stress Test Kit (Seahorse Bioscience, Agilent) according to manufacturer’s protocol.

**RNA stability assay**

To measure the RNA stability, GBM cells were treated with actinomycin D (ActD, 2 μg/ml) for indicated time (0 h, 3 h, 6 h). Then, total RNA was isolated and detected using quantitative real-time PCR for the relative level normalized to β-actin.

**MeRIP-quantitative real-time PCR (MeRIP-qPCR)**

MeRIP-qPCR was performed according to previously published literature with modification. In brief, total RNA was isolated and fragmented to 100 nt RNA. With the addition of ZnCl2 and anti-m^6^A polyclonal antibody (Synaptic Systems, 202003), protein A/G magnetic beads (Thermo Fisher Scientific) were used to capture the immunoprecipitated RNA fragments at 4 ℃. The precipitation was eluted from the beads with immunoprecipitation buffer and the HK2 mRNA concentration was measured with quantitative real-time PCR.

**RNA-binding protein immunoprecipitation (RIP)**

U251 cells with stably CASC9 overexpression and U87MG cells with stably CASC9 knockdown were collected and lysed with radioimmunoprecipitation (RIP) lysis buffer (Magna RIP Kit, Millipore, MA). After sonication, immunoprecipitations was performed using anti-IGF2BP2 antibody (Abcam, ab128175, 1:1000) and control rabbit IgG antibody overnight at 4 ℃. After washing, RNAs were extracted from the immunoprecipitated RNA-protein complex was detected by quantitative real-time polymerase chain reaction (qRT-PCR). The relative data was normalized to input.

**Statistical analysis**

All data in this paper was statistically analyzed by GraphPad 7.0 and SPSS 20.0. One-way ANOVA test was performed for various group and t-test was performed within two-independent group. Patients’ survival was calculated using Kaplan-Meier curves following log-rank test analysis. p<0.05 was thought as statistical significance.
