# Supplementary material for: m6A reader IGF2BP2-stabilized CASC9 accelerates glioblastoma aerobic glycolysis by enhancing HK2 mRNA stability
Source: Cell Death Discov. 2021 Oct 13;7:292. doi: 10.1038/s41420-021-00674-y (PMC8514511; doi:10.1038/s41420-021-00674-y)
Supplement: Supplementary file 3 — Table S1 [file 41420_2021_674_MOESM3_ESM.docx]

**supplement Table S1**. Primers sequences for qRT-PCR and sequences of shRNA.

|  | Sequences |
| --- | --- |
| CASC9 | forward, 5’-TTGGTCAGCCACATTCATGGT-3’  reverse, 5’-AGTGCCAATGACTCTCCAGC-3’ |
| IGF2BP2 | forward, 5’-AGTGGAATTGCATGGGAAAATCA-3’  reverse, 5’-CAACGGCGGTTTCTGTGTC-3’ |
| HK2 | forward, 5’-GAGCCACCACTCACCCTACT-3’  reverse, 5’-CCAGGCATTCGGCAATGTG-3’ |
| shRNA-CASC9-1 | 5’-GCCUGUGAUAGCAGAACAATT-3’ |
| shRNA-CASC9-2 | 5’-GGGCAUUGAGAAGUUAGAATT-3’ |
| shRNA-CASC9-3 | 5’-GGACUCAUAUUACCAGUCUTT-3’ |
| beta-actin | forward, 5’-CTCCATCCTGGCCTCGCTGT-3’  reverse, 5’-GCTGTCACCTTCACCGTTCC-3’ |
